# Body Size Differences between Foraging and Intranidal Workers of the Monomorphic Ant *Lasius niger*

**DOI:** 10.3390/insects11070433

**Published:** 2020-07-10

**Authors:** Mateusz Okrutniak, Bartosz Rom, Filip Turza, Irena M. Grześ

**Affiliations:** 1 Department of Zoology and Animal Welfare, University of Agriculture in Krakow, al. Mickiewicza 24/28, 30-059 Kraków, Poland; bartek1506@vp.pl (B.R.); irena.grzes@urk.edu.pl (I.M.G.); 2 Institute of Environmental Sciences, Jagiellonian University, ul. Gronostajowa 7, 30-387 Kraków, Poland; filipturza@gmail.com

**Keywords:** ants, body size, monomorphism, task division

## Abstract

The association between the division of labour and worker body size of ants is typical for species that maintain physical castes. Some studies showed that this phenomenon can be also observed in the absence of distinct morphological subcastes among workers. However, the general and consistent patterns in the size-based division of labour in monomorphic ants are largely unidentified. In this study, we performed a field experiment to investigate the link between worker body size and the division of labour of the ant *Lasius niger* (Linnaeus, 1758), which displays limited worker size variation. We demonstrated that the body size of workers exploring tuna baits is slightly but significantly smaller than the size of workers located in the upper parts of the nest. Comparing the present results with existing studies, large workers do not seem to be dedicated to work outside the nest. We suggest that monomorphic workers of certain body sizes are flexible in the choice of task they perform, and food type may be the important determinant of this choice.

## 1. Introduction

Division of labour is one of the most fundamental features of social insect colony behavior. It was found that task allocation may contribute to the evolutionary success of social insects [1,2,3]. A number of studies confirmed that division of labour is related to the worker body size of many social insects, such as bees [4], bumblebees [5,6,7], eusocial wasps [8], termites [9] and especially ants [10,11,12]. Describing the proximate factors determining the association between body size and the work organization of social insects may lead to a better understanding of the evolution of their phenotypic diversity.

The relationship between body size and task allocation of ant workers is mostly recognized from studies of species with discrete worker subcastes (polymorphic ants) [13,14]. The most complex system of work organization is known for Attini ants. A striking example of task partitioning is observed in the fungus-growing ant *Atta cephalotes*, which has an extremely size-diversified worker caste [15,16,17,18]. The less radical form of size-related division of labour is the diversification into minor and major workers. In many dimorphic species of the genus *Pheidole*, major workers showed a trend toward specialization for defence and/or food storage while minor workers perform virtually all tasks, including nest maintenance, brood care, and foraging [19,20].

Polymorphic ants are commonly investigated in the context of the division of labour, however, the vast majority of ants do not exhibit well-defined physical castes [19]. The size of workers of so-called monomorphic ants typically follows a normal distribution and its range is narrower than that of polymorphic ones. Although the body size of monomorphic ants is not expected to be associated with the division of labour, it was shown that in some species, workers of different sizes also prefer to perform some tasks over others [21,22,23,24]. In *Lepthotorax longispinosus,* workers that forage for food were the largest individuals available, while the ants engaged in social interactions such as carrying pupae were relatively small [21]. Thus, for ants, a size-dependent division of labour seems to be a general phenomenon, which also encompasses species with a small size variation. However, knowledge on the size-dependent division of labour of monomorphic species is largely based on limited data and therefore does not allow the general patterns of work division to be well grounded. To our best knowledge, the size-based division of labour was investigated only for a few monomorphic species, i.e., *Lepthotorax longispinosus*, *Temnothorax rugatulus*, *Lasius niger*, *Formica polyctena*, and the question of its advantage for colony functioning is still unanswered [21,22,23,24]. To identify general trends in size-dependent division of labour, the important question can be asked if the results of studies are consistent and repeatable across species, seasons or experimental set-ups. 

Here, we present a simple field experiment on the association between body size and the division of labour of the common garden ant *Lasius niger* (Linnaeus, 1758). In our previous study of this species, we demonstrated that workers foraging for honey are larger than domestic ones [23]. In this study, we checked whether the preference of large workers to forage over performing tasks in the nest holds true when using tuna baits instead of honey baits. We compared the body size of *L. niger* workers attracted to tuna with that of workers sampled directly from the nest (intranidal, nurses). We expected a similar result to our previous experiment, which would suggest the repeatable tendency of large *L. niger* workers to forage. We discussed the limitations of the present methods and assumptions.

## 2. Materials and Methods

### 2.1. Study Species

The black garden ant (*Lasius niger*) is one of the most common species in the Palearctic, with unusually broad ecological flexibility. *Lasius niger* prefers open and sunny habitats, including various anthropogenic environments such as meadows, pastures and green spaces in urban areas. *Lasius niger* is monomorphic (no morphologically distinguishable subcastes of workers) and strictly monogynous (each colony has one queen). Mineral nest mounds usually contain from 100 to more than 10,000 workers. Main nuptial flights take place from July to late August. The species of interest is both aphidicolous and carnivorous [25]. Its diet consists of honeydew from aphids, plant nectar as well as small insects [25,26,27].

### 2.2. Sampling

**Figure 1:**
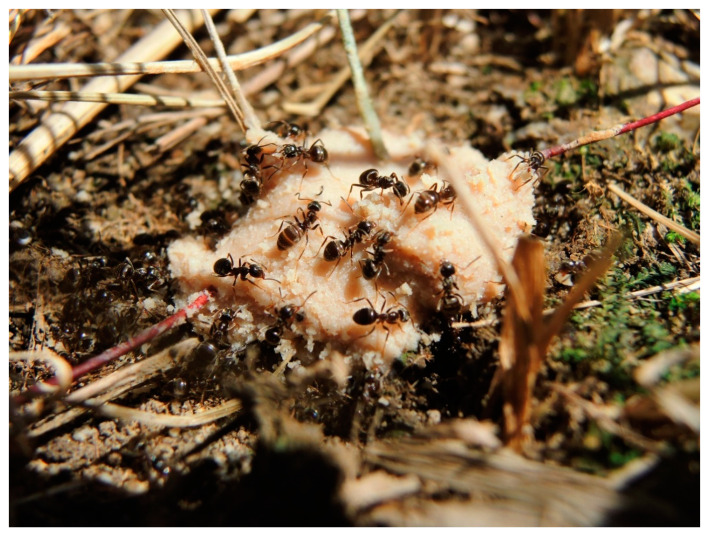
Workers of *Lasius niger* attracted to tuna bait (phot. M.O.).

*Lasius niger* workers were collected during two subsequent days at the beginning of July 2016 from one meadow in southern Poland. Sampling was performed between 9 a.m. and 12 a.m. The temperature during 2 days of sampling reached a maximum of 21 °C and 26 °C, respectively. The sampling included 30 independent mature colonies (checked for the presence of male or female pupae). The diameter of each nest ranged from 40 to 60 cm and the distance between the colonies was greater or equal to 3 meters. Two independent samples of workers were collected from each colony: one of them consisted of workers attracted to tuna bait, and the other consisted of intranidal workers, i.e., sampled from the upper nest chambers. Tuna baits were placed at a distance of 50 cm from each nest. Each tuna bait had a fresh mass of about 3.5 g. After 15 (±3) min, almost all workers attracted to the tuna bait (about 50) were collected using a plastic exhauster (Figure 1). The dynamics of the foragers arriving at food described by Chadab and Rettenmeyer shows that after 15 min of putting out bait, an abundant sample of ants can be collected [28]. Finally, about 50 intranidal workers were collected after opening the nest from the depth where the first sexual appeared, i.e., approximately 20 cm. Because intranidal workers were sampled from the chambers containing larvae and pupae, the intranidal workers most probably constituted nurses. Workers were preserved in 70% ethanol until being measured. Species identification followed Czechowski et al. [25].

### 2.3. Morphological Measurements

The body size of each ant was measured, determined as the maximum head width above the eyes (“HW” according to Czechowski et al. [25]). Head width is a commonly used estimator of the body size of ants, including *Lasius* ants [29,30,31,32]. The measurements were performed under 100 magnification using a Met-153 metallographic microscope connected to a digital camera. Head widths were measured to the nearest 0.00001 mm based on digital photos using the Panasis v2.4.2 Huvitz program. All measurements were made by one person (MO). Each sample of intranidal workers (I-workers) numbered 50 individuals, while the samples of bait-attending workers (T-workers) ranged between 32 and 50 individuals. A total of 2905 measurements were made. In order to check the accuracy of the measurements, 25 ants were measured twice. The results of the first and the second set of measurements were correlated using simple regression. The high correlation coefficient (r = 0.99, *p* < 0.0001) indicates a satisfactory repeatability of the measurements.

### 2.4. Statistical Procedures

In order to test the difference in mean body size between *Lasius niger* foraging workers attracted to tuna baits (T-workers) and workers collected directly from the nest (I-workers), a two-way ANOVA was applied. “Head width” was used as the dependent variable, while “colony” (N = 30) and “worker class” (T-workers and I-workers) as independent variables (factors). The interaction between “colony” and “worker class” was also included. The residuals were normally distributed (Shapiro–Wilk test, *p* = 0.61). All statistics were calculated using Statgraphics Centurion vXVIII (StatPoint Technologies, Inc. 2019) and Prism 8.3 (GraphPad Software, San Diego, CA, USA).

## 3. Results

**Figure 2:**
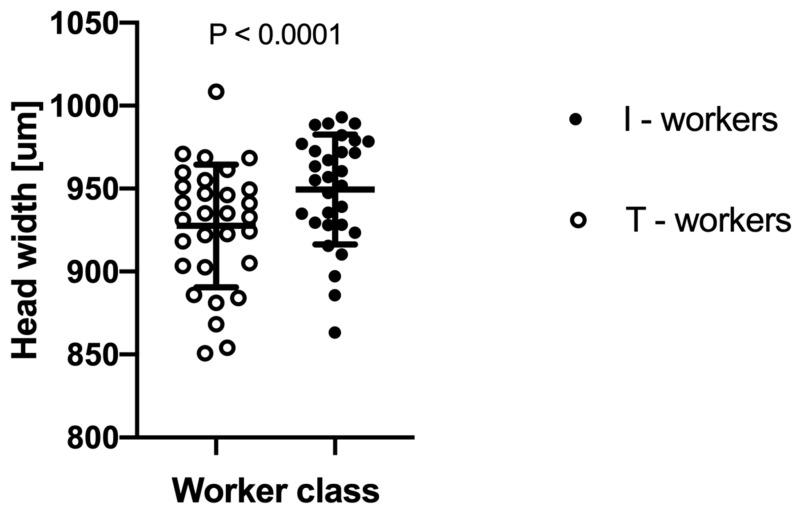
The difference in mean head width between *Lasius niger* foraging workers attracted to tuna baits (T-workers) and workers collected directly from the nest (I-workers). Each dot indicates an average head width within a given colony. Whiskers denote standard deviations (SD).

**Figure 3:**
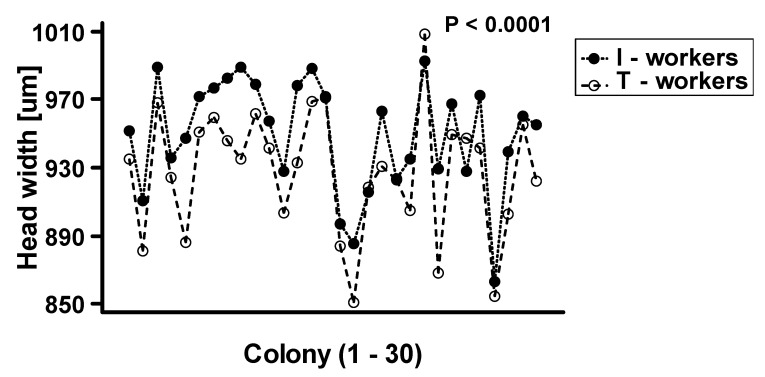
The plot of interaction between worker class and colony (N = 30) on the mean body size of *Lasius niger*. T-workers-foraging workers attracted to tuna baits, I-workers-workers collected directly from the nest.

The two-way ANOVA analysis showed that both factors—“colony” and “worker class”—as well as their interactions, are significant at *p* < 0.0001 (Table 1). We observed that intranidal workers were generally significantly larger than workers attracted to the tuna (Figure 2). However, as the interaction between “colony” and “worker class” was highly significant at *p* < 0.0001, the relative differences in body size between worker classes vary among the colonies (Figure 3).

## 4. Discussion

The aim of this study was to compare intranidal workers (collected directly from the nest) with foragers attracted to tuna baits placed outside the nests. We found that foragers were slightly but significantly smaller than intranidal workers. Surprisingly, this result contradicts the result of our previous study showing that if we applied a similar experimental setup but with honey baits, the foragers were larger than the intranidal ants [23]. Therefore, we do not confirm the repeatable tendency that large workers of *L. niger* prefer tasks outside the nest.

In order to explain why workers foraging for tuna are smaller than intranidal workers, we propose a hypothesis that the size of the workers involved in foraging for certain food types is associated with within-species competition. It was proven by many authors that the foraging decisions of *L. niger* are altered by food type, sucrose concentration, food volume and quality [33,34,35,36]. In the field, protein food is scattered and ephemeral in time, while aphid colonies, the sources of carbohydrates, are spatially stable and are highly attractive to ants. Therefore, carbohydrates potentially require *L. niger* workers to undertake considerable effort in defending them against potential competitors [37,38]. In turn, Kay and Rissing [39] found that the small workers of the monomorphic ant *Formica perpilosa* avoid competitive, novel environments in contrast to their large workers [39]. Therefore, it is possible that the small workers of *L. niger* tend to forage for tuna because the probability of competition may be lower in comparison with honey. We have to admit that the proposed explanation may not apply to other monomorphic ants. As suggested by Véle and Modlinger [24], the function of the large workers of *Formica polyctena* is associated with their effectiveness in defending and maintaining the nest [24].

We have to admit that a direct comparison of these findings should be done with attention. In both experiments, we used a similar design, but seasons and sites differ between the studies. Therefore, the inconsistency of the results may be explained by the difference in food type used to attract the ants, but also by unmeasured environmental factors like colony size, age or temperature. On the other hand, the idea that food type may influence the choice of the task is supported by the findings of Véle and Modlinger [24]. They proved that the size of *Formica polyctena* workers affects their work division; ants attracted to tuna differed significantly in head width than the ants attracted to honey. Independently of the proximate factors explaining the inconsistency of the results, one can conclude that *L. niger* ants have no settled body size for foragers. Thus, the tendency of larger workers to forage outside the nest, similar to that observed in weakly dimorphic species, may not be the rule for monomorphic ants. Gaining a deeper understanding of the body-size dependent division of labour in monomorphic ants would require advanced methods, for example, marking the foragers in the field in order to follow the individual decisions on the task to be performed. Further studies are needed to establish whether the body size of foragers attracted to certain types of bait changes with the time of the bait exposure. Because thermal tolerance may differ between large and small workers [11], it may be expected that the body size of foragers is a function of changes in external temperature during the day. Essentially, the present study only investigated the relationship between body size and task allocation, but did not evaluate the adaptive value of the observed relationships. Consequently, the question on the effectiveness of workers of a certain size in performing the task they choose is still open.

## 5. Conclusions

In conclusion, this study indicates that the division of tasks of the ant *Lasius niger* is related to worker body size but it simultaneously shows that larger workers are not specialised in foraging. It is likely that when compared to polymorphic ants, monomorphic workers of a certain size are more flexible in the choice of the task they perform. Comparing our results with the studies of other authors, food type may be an important determinant of task choice. This result should encourage further analysis on the factors determining the size-dependent division of labour in species having narrow size variation.

## Figures and Tables

**Table 1:** Results of the two-way ANOVA for *Lasius niger* worker size. “Head width” was used as the dependent variable while “colony” and “worker class” were the independent variables (factors). The interactions among the factors is also presented.

Source	Df	F-Ratio	*p*-Value
Main Effects			
Colony	29	51.36	*p* < 0.0001
Worker Class	1	162.7	*p* < 0.0001
Interaction			
Colony*Worker Class	29	4.294	*p* < 0.0001
Residual	2845		
